# Pilot Polygenic Risk Score in Health and Aging Brain Study ‐ Health Disparities (HABS‐HD) Mexican Americans

**DOI:** 10.1002/alz.095129

**Published:** 2025-01-09

**Authors:** Mohammad Housini, Neetesh Pandey, Giuseppe Tosto, Robert Barber, HABS‐HD Study Team

**Affiliations:** ^1^ University of North Texas Health Science Center, Fort Worth, TX USA; ^2^ Columbia University, New York, NY USA; ^3^ Taub Institute for Research on Alzheimer’s Disease and the Aging Brain, Vagelos College of Physicians and Surgeons, Columbia University, New York, NY USA; ^4^ Department of Neurology, Vagelos College of Physicians and Surgeons, Columbia University, and the New York Presbyterian Hospital, New York, NY USA; ^5^ Institute for Translational Medicine, Fort Worth, TX USA

## Abstract

**Background:**

Alzheimer’s Disease (AD) is a complex disease with varying etiology based on genetic, environmental, and social factors. Most of the literature in the field has focused on studying AD in individuals with European ancestry. Recent efforts have shown that AD and related diseases show differences based on ethnicity. Our previous work has demonstrated variable allele frequency in the top ten risk alleles between Mexican American and Non‐Hispanic White Americans in the Health and Aging Brain Study‐ Health Disparities (HABS‐HD). It is essential to study AD in minorities, who are estimated to experience significantly increased disease burden in the next few decades.

**Method:**

To expand our previous work, we conducted a pilot Polygenic Risk Score (PRS) study on N = 665 Mexican Americans from the HABS‐HD Study with complete phenotype data. Using quality controlled and cleaned genotype data from these individuals, PRSice was used to construct a PRS model using age, gender, APOE E4 status, and the first 5 Principal Components as covariates. Generalized Linear Regression was used to predict association between the PRS results and the predicted phenotype (AD). AUC was calculated for both the APOE E4‐only model and the full model.

**Result:**

The pilot PRS model performed well on N = 665 Mexican Americans from HABS‐HD, with a R^2 best fit at P – value threshold = 0.01555. The ROC graph shows AUC of 0.605 for PRS with covariates and 0.54 without covariates.

**Conclusion:**

Our pilot study shows that a PRS produced for this population can accurately predict disease phenotype. We plan to conduct larger examinations of this nature and further elaborate on minority health disparities in order to fill the gaps in the literature and expand our understanding of genetic risk in various ethnic groups.

